# The effect of Community Based Education and Service (COBES) on medical graduates’ choice of specialty and willingness to work in rural communities in Ghana

**DOI:** 10.1186/s12909-016-0602-8

**Published:** 2016-03-01

**Authors:** Anthony Amalba, Walther Nicolaas Karel Anton van Mook, Victor Mogre, Albert Jakob Johannus Antonius Scherpbier

**Affiliations:** University for Development Studies (UDS), School of Medicine and Health Sciences (SMHS), Tamale, Ghana; School of Health Professions Education, Faculty of Health, Medicine, and Life Sciences, Maastricht University, Maastricht, The Netherlands; Department of Health Professions Education and Innovative Learning, School of Medicine and Health Sciences, University for Development Studies, P. O. Box 1883, Tamale, Ghana

**Keywords:** Community-based education, Choice of specialty, Rural placement, Medical graduates, Service, Career choice, Community

## Abstract

**Background:**

Career choices and placements of healthcare professionals in rural areas are a major problem worldwide, and their recruitment and retention to these areas have become a challenge to the health sector. The purpose of this study was to investigate the effect of Community Based Education and Service (COBES) on medical graduates' choice of specialty and willingness to work in a rural area.

**Method:**

This cross sectional survey was conducted among 56 pioneering graduates that followed a Problem Based Learning/Community Based Education and Service (PBL/COBES) curriculum. Using a mixed methods approach, open-and closed-ended questionnaire was administered to 56 graduates. Cross tabulation using Chi-square test were used to compare findings of the quantitative data. All qualitative data analysis was performed using the principles of primary, secondary and tertiary coding.

**Results:**

All 56 graduates answered and returned the questionnaire giving a 100 % response rate. 57.1 % (32) of them were male. Majority of them lived in towns (41.1 %) and cities (50 %) prior to medical school. A significant number of graduates (53.6 %,) from the cities, without any female or male predominance said COBES had influenced their choice of specialty. Again, a significant proportion of graduates from the towns (60.9 %,) and cities (67.8 %,), indicated that COBES had influenced them to work in the rural area. However, there was no significant difference between males and females from the towns and cities regarding the influence of COBES to work in the rural area.

Qualitative data supported the finding that COBES will influence graduates willingness to work in the rural area

**Conclusion:**

The majority of graduates from the towns and cities in Ghana, with a male predominance, indicated that COBES may have influenced their choice of specialty and willingness to practice in the rural areas despite their town or city based upbringing.

**Electronic supplementary material:**

The online version of this article (doi:10.1186/s12909-016-0602-8) contains supplementary material, which is available to authorized users.

## Background

Recruitment and retention of healthcare professionals in the rural areas are major problems worldwide especially in developing countries. There is some evidence that Community- Based Education and Service (COBES) can be used to prepare and acclimatise healthcare professionals to work in rural areas and bring equity in the distribution of health professionals to benefit the rural communities [1, 2]. Attention has now been focused on education and retention of medical doctors in Africa. The most commonly reported strategies to improve retention include increasing salaries for faculty, strengthening post-graduate education and launching or strengthening community-based education programmes [3]. Despite the challenges of COBES, such as unreliable public utilities, language barriers, the maintenance of high educational standards with the community doctors/health workers who supervise learners, the advantages of COBES include lower attrition rates, greater perceived ability to function in rural community and high satisfaction expressed by students and community members alike [4]. Structured community exposure and community-based education provide students with experiences of working with underserved populations and improve graduates’ preparation to deal with national health problems [5].

In Ghana, most of the communities are considered as rural. Only localities equal to or exceeding 5,000 persons have been classified in Ghana as urban since 1960. Despite the growth of urban population , Ghana continues to be a nation of rural communities, with the rural areas representing an estimated 66 percent of the population [6]. The physician to population ratio in the Greater Accra region is 1:5,000, whereas in the largely rural Northern region, with a population of over 2 million people, it is 1: 92,000 [7]. This skewed distribution has consequences on the quality and availability of health care in remote regions of the country [8].

Socio-economic conditions negatively impact on the willingness of healthcare professionals to work in rural areas in the developing countries. The inequity in distribution of health professionals, especially doctors, between the urban and rural areas according to studies conducted in Ghana have been attributed to better social amenities, infrastructure, income, an opportunity for career progression that the cities offer compared to the rural communities. Lack of recognition, rewards, mentoring, continuous education and the occurrence of professional imprisonment are commonly cited as reasons for the low rate of willingness to accept or seek rural postings [8–11]. Some authors therefore suggest to include a compulsory student rotation in rural areas to decrease or remove the often voiced fears perceived by medical students (often from urban areas) and better inform them about the actual practice conditions in remote rural areas [9].

Students’ rural background (those who are born and grow up in the rural areas) has been found to be associated with future rural career choice. This is evidenced by the results of a considerable number of studies originating in America [12], Australia [13–15], Canada [16], Japan [17], Norway [18], South Africa [19] and Scotland [20]. Without exception, these studies have confirmed that medical students from a rural background are more likely to take up rural medical practice than their peers from city origins.

In contrast, a limited number of studies on factors which influence career choices of trainees in rural areas of low income countries are available. A recent review of attraction and retention policies highlighted the need to analyse first, local data about health worker decision making and the challenges of rural service in a given country in order to get information about the value of various incentives [8]. Most of the studies conducted in rural settings have been situated in developed countries [21]. However, we have to be mindful that the definition of ‘rural’ can vary and rural areas have distinct characteristics that include isolation, limited access to healthcare, small populations, significant distances between services and providers and informal social structures [22]. Though rural practice comes with its unexpected challenges regardless of the geographical location, rural settings in the developed world can be totally different from rural settings in the developing world. Notable differences are unmotorable road network, lack of electricity, and more prominent lack of social infrastructure (e.g. health facilities and schools). Studies performed in more developed countries are therefore difficult to compare to those performed in developing countries.

Up to date, the Ghana Ministry of Health (MoH) and donor agencies remain uncertain about which investments have the potential to measurably improve the number, retention and distribution of health personnel. Investments have been cautious and creating sufficient human resource for health has been described as a potential challenge until interventions have been rigorously evaluated for the desire impact in areas that could improve the number, retention and distribution of health personnel [8, 23, 24].

While previous research has thus looked at working conditions of health workers and incentives to promote uptake of rural posts, disparities or gaps regarding health professionals especially doctors still exist between the rural and urban settings. Though few studies in Ghana have looked at the factors and likelihood or willingness of Ghanaian medical students to practice in the rural area, the effect on the use of rural communities as a training platform on graduates choice of specialty and practice location in Ghana has not been investigated [8]. Nevertheless, most of the literature acknowledged the need to use rural communities in the training of medical students as well as the need to compulsorily define the period of this rural education and service [8–11].

Since evidence for this approach is thus largely lacking, this study explores whether the exposure of medical graduates to the rural communities as part of their training has an effect on their choice of specialty and willingness to work in the rural area.

## Methods

### Setting

The University for Development studies (UDS), School of Medicine and Health Sciences (UDS-SMHS) which was established in 1996, is one of the five campuses of UDS and located in Tamale in the rural Northern Region of Ghana. UDS-SMHS adopted a (PBL/COBES) medical school curriculum in 2007 in response to reforms in medical education. The COBES component of the PBL/COBES curriculum is a platform that enables students to learn and also provide service to the community. After a first year of participating in the University-wide interfaculty community-based programme, the COBES programme for medical students starts in year 2 and continues up to year 7. For the first 3 years (year 2 to 4), students are sent to communities with at least a primary health care facility. Each of these three years, in the period from July to August, the students spend four weeks in the community in groups of 8-10 students per community. The COBES curriculum is iterative and each year builds upon the previous years’ experience thereby updating, improving and expanding the activities of the previous year. Depending on the year of the programme students are expected to identify and explain factors (e.g. demographic, economic, social, cultural, political and environmental) that affect the community health, perform a study resulting in a community health diagnosis to identify community health needs and subsequently prioritise them and identify the resources available in the community to contribute to meeting those needs. At community level students thus design and implement a health intervention based on community health diagnosis. They also take turns to rotate through the various sections of the health facility, like, for example, dispensary, consulting rooms, Maternal and Child Health clinic and the laboratory as well as participate in the scheduled immunizations by the health facility in the communities. In years 5, 6 and 7 students are sent to district hospitals to introduce them to the secondary level of care, again for a period of 4–6 weeks.

### Participants and questionnaire

Following a cross-sectional design, this study was conducted from June to July 2014. Participants were medical graduates of the UDS-SMHS. These graduates were the pioneers (the first batch of students to follow the programme) of the PBL/COBES curriculum. They had finished their educational program at UDS-SMHS and were leaving the school to various accredited hospitals for their internship training.

Using trained research assistants, all recruitment and data collection processes were conducted during an orientation program that sought to prepare the graduates for their internship training at various hospitals in the country. Although, 78 students graduated, only 56 of them attended the orientation programme. Thus the rest of the 22 graduates did not participate in the study because they got the information for the orientation programme late and as a result did not tend up for the programme. Given the fact that the students’ had graduated, the orientation programme was the only opportunity to administer the questionnaire to them. All data was collected using a 14-item questionnaire that consisted of opened- and closed-ended questions. The questionnaire assessed demographic factors, the medical graduates’ perception of the usefulness of COBES and the perceived influence of COBES on their choice of specialty and willingness to accept rural postings. The setting of the residence of the medical graduates prior to admission to the medical school was also assessed (see Additional file 1). The items of the questionnaire were derived from the literature and assessed by experts in the field who considered them to be content valid. To ensure comprehensibility and understanding, the questionnaire was pretested on a sample of five students, the results of which led to slight modifications of the items. Ethical approval was granted by the Ethics Committee of the School of Medicine and Health Sciences, University for Development Studies.

### Data analysis

Quantitative data were entered into Microsoft Excel and analysed using GraphPad Prism, Version 5.01 (GraphPad Software Inc., San Diego CA). Results were presented as frequencies and proportions of the total sample recruited. Association between variables such as gender, place of residence, choice of specialty and practice location were assessed using cross tabulation and Chi-square test. The setting of the residence of the medical graduates prior to admission was classified into three categories: villages, towns and cities. All residential settings having populations less than 2000 people, not classified as urban and lacking basic infrastructures like electricity, potable drinking water, public utilities were considered as villages. Place of residents classified as urban and having population less than 100, 000 people was classified as a town. These had better public utilities than the villages. All place of residents that were urban and had settlements of 100, 000 people and more and may span municipality or entity boundaries were classified as cities. All of these definitions were based on the UNDP’s definitions of villages, towns and cities [25].

Qualitative data analysis of responses to opened-ended questions was performed using Atlas ti version 6.0.15 GmbH-Berlin, applying the principles of primary, secondary and tertiary coding [26]. The responses to all the opened-ended questions were entered into Microsoft-word with the help of a research assistant. The responses were read independently by AA and VM identifying common themes through the constant comparison method, identifying trends and using the common opinions expressed by the graduates. The identified themes were independently coded, enabling us to compare between graduates’ responses. The independent codes generated by AA and VM were cross-checked by the second and fourth authors (AS and WvM). All discrepancies in the process were discussed until consensus was reached. Illustrative quotes were presented to underscore the findings where applicable.

## Results

This section consecutively discusses the numerical results of the questionnaires and the results of the qualitative data analysis of answers to open-ended questions. Appropriates quotes from the graduates are cited.

### The quantitative results of the student questionnaire

All 56 graduates answered and returned the questionnaire giving a 100 % response rate. As shown in Table 1, the majority of the graduates were males (57.1 %, *n* = 32), lived in cities (50.0 %, *n* = 28) and perceived that COBES will influence their choice of specialty (44.6 %, *n* = 25). Although the differences were not significant, a higher proportion of females (50 %, *n* = 12) than males (40.6 %, *n* = 13) said COBES will influence their choice of specialty. Sixty-four percent of the graduates indicated that COBES will influence their willingness to work in a rural location and were significantly more likely to be male graduates (78.1 %, *n* = 25) than females (45.0 %, *n* = 11). Irrespective of gender differences, 82.1 % (*n* = 46) of the students said COBES will be useful for their future practice as doctors.

**Table 1:** Demographic characteristics, place of residence and graduates perceived influence of COBES on choice of specialty, willingness to practice in a rural setting and perceived usefulness of COBES

	Total (*n* = 56)	Male (*n* = 32)	Female (*n* = 24)	*p*-value
Place of Residence				
Village	5(8.9 %)	5(15.6 %)	0(0.0 %)	0.0638
Town	23(41.1 %)	12(37.5 %)	11(45.8 %)	0.590
City	28(50.0 %)	15(46.9 %)	13(54.2 %)	0.788
Choice of specialty				
Will affect	25(44.6 %)	13(40.6 %)	12(50.0 %)	0.589
Will not affect	23(41.1 %)	14(43.8 %)	9(18.8 %)	0.788
Unsure	7(12.5 %)	5(15.6 %)	3(12.5 %)	0.451
Choice of practice in a rural area				
Will affect	36(64.3 %)	25(78.1 %)	11(45.0 %)	0.023
Will not affect	12(21.4 %)	4(12.5 %)	8(33.3 %)	0.099
Unsure	8(14.3 %)	3(9.4 %)	5(20.8 %)	1.000
Perceived usefulness of COBES				
Useful	46(82.1 %)	28(87.5 %)	18(75.0 %)	0.298
Not Useful	10(17.9 %)	4(12.5 %)	6(25.0 %)	

Graduates perceived that influence of COBES on their choice of specialty and willingness to work in a rural location was stratified by place of usual residence and presented in Table 2. Twenty percent of graduates from villages, 39.1 % from Towns and 53.6 % from cities said COBES will influence their choice of specialty. The differences were not statistically significant. Although the differences were not significant a higher proportion of graduates who lived in cities (67.9 %, *n* = 19) compared to those who lived in villages (60.0 %, *n* = 3) and towns (60.9 %, *n* = 14) said COBES have influenced their willingness to work in a rural location.

**Table 2:** Graduates perceived influence of COBES on choice of specialty and practice location stratified by place of residence

Variable	Village (*n* = 5)	Towns (*n* = 23)	Cities (*n* = 28)	*p*-value
Choice of specialty				
Will affect	1(20.0 %)	9(39.1 %)	15(53.6 %)	0.299
Will not affect	2(40.0 %)	11(47.8 %)	10(35.7 %)	0.681
Unsure	2(40.0 %)	3(13.0 %)	3(10.7 %)	0.208
Choice of practice in a rural area				
Will affect (*n* = 36)	3(60.0 %)	14(60.9 %)	19(67.9 %)	0.855
Will not affect (*n* = 12)	1(20.0 %)	5(21.7 %)	6(21.4 %)	0.996
Unsure (*n* = 8)	1(20.0 %)	4(17.4 %)	3(10.7 %)	0.739

### The qualitative results of the medical graduates’ questionnaire

The responses to all the open-ended questions were typed into Microsoft-word with the help of a research assistant. The responses were read through independently by AA and VM identifying common themes through the constant comparison method, identifying trends and using the common opinions expressed by the graduates. The identified themes were independently coded, enabling us to compare between graduates’ responses. The independent codes generated by AA and VM were cross-checked by the second and fourth authors (AS and WvM). All discrepancies in the process were discussed until consensus was reached. Illustrative quotes were presented to underscore the findings where applicable.

### Adaptation to rural lifestyle

COBES develops graduates to adapt to rural lifestyle making it easy to accept to work in the rural areas. *‘I am more willing to work in a rural area, although I have always wanted to work in the north, I now have the motivation to do so’ (City, female).*

### Equity to healthcare

During the period of their COBES program graduates realized the poverty levels in the communities and the need to help the less privileged to get access to quality and equity in health care. *‘It (COBES) created awareness on the plight of rural northern Ghana. I will not refuse a posting to northern Ghana’ (City, male),*

### Community health needs

The community health needs and the limited human resource in the community invoke the willingness and preference of graduates to want to work in the rural areas *‘I am willing to work in the district and extend to the deprived areas of Ghana because that is where the real problems are’ (Town, male).*

### Hospitality and culture of community members

Graduates learn the culture and lifestyle of the community when they interact with community members. As they live and work in the community without any friction, coupled with the hospitality of the community members, their willingness to go back to the community after graduation becomes strong. *‘My experience in COBES has increased my desire to work in rural areas provided I am assured of continuous professional development (Town, male)’*

## Discussion

This study revealed that the graduates from the towns and cities perceive that COBES could influence their choice of specialty with an overwhelming majority of them willing to work in the rural areas. Furthermore, COBES was shown to predominantly affect males regarding the willingness to work in rural areas. In contrast to the first finding, the latter finding that COBES has more effect on males than females regarding the willingness to work in rural areas was not unexpected. There is evidence that medical students from rural background are more likely to take up rural medical practice than their peers from the cities [12]. However, this study conversely shows that when students from the towns and cities have part of their training in the rural community, it has some influence on their choice of specialty and preference to work in the rural communities [9]. When searching the literature to find support for this less well- known association, a similar study in Australia provided the needed support. The study was performed among graduates following a Parallel Rural Community Curriculum (a community based medical education program where students spend one academic year in the community) and revealed that this extended rural undergraduate experience has influenced them to undertake a rural career path, despite a city based upbringing [27].

The present and prior findings are thus supportive of the notion that adaptable variables such as educational experiences can have a significant reinforcement effect as well as a positive influence on the practice intentions of medical students as they progress from matriculation to graduation. In addition to individual student characteristics including students’ social background and geographical location of upbringing, participation in community health, cultural awareness/diversity and language-learning, educational experiences were associated with intention to practice in underserved areas [28].

Although, rural origin has a strong motivating factor for students to return to rural practice in the developed world and has been factored into their selection and admission policies, this has not been included in the selection and admission policy of most developing countries including Ghana, either because of lack of enough evidence of this factor as a strong motivation to return doctors and other healthcare professionals to the rural areas after graduation or a very strict entry grade point to universities in developing countries. In the light of these findings, and the international literature, it would be appropriate that Ghana takes a critical look at the 'selection and admission policies in selecting students into the medical schools of the country.

A second important finding was that COBES continues to have more influence on males than female as it was clearly shown that 80 % (*n* = 12) (Table 1)of male graduates indicated that COBES will influence them to work in a rural and 87.5 % (*n* = 28) (Table 1) reporting COBES to be very useful to them. This has been reported in similar studies conducted in Australia and other countries, that men were more likely than women to enter rural practice [12, 29, 30]. However, there was similar agreement among males and females on the influence of COBES regarding their choice of specialty. This was similarly reported in a study on the role of intrinsic versus extrinsic motivations on medical students’ willingness to work in rural areas in Ghana [9]. The study also found that the female gender was strongly associated with reduced interest in rural practice. This was also similarly reported among health staff that women are less likely to accept postings in remote areas [31, 32]. Some of the explanations of reduced interest among women to practice in rural areas were for varying family reasons: they prefer to live in the same geographical area in which their husbands worked, have difficulty of convincing their husbands to get transfers and following their wives to the rural areas, and desire better education, than can be found in the rural areas, for their children.

So far, strategies to recruit and retain rural health workers vary widely, including education reforms (conducting training programs in rural areas), financial incentives, and compulsory service. Evidence of the impact of these strategies especially of educational reforms on health worker distribution in low-and middle-income countries (LAMICs) is poor, due to structural obstacles within the health system, lack of legislation to back up reform policies, and resistance to change, which challenge the systematic implementation of policy change and evaluation of impact [11].

The Ghana Ministry of Health (MoH) has implemented a number of incentives aiming at recruiting and retaining health staff in the country and deprived areas. These included a 20-30 % salary top up for health staff in deprived areas (implemented in 2004) and a staff vehicle purchase scheme (implemented in 1997) [8]. Neither has however yielded the desired results in addressing the lack of health professionals in remote areas.

Curriculum planners could also learn from this study and acknowledge that establishment of COBES as part of health training institutions curricula to provide rural exposure also serves the purpose of motivating students and creating a favourable attitude towards rural practice and positively influences graduates on their choice of specialty and willingness to work in the rural areas.

### Limitations of the study

The purposeful selection of participants may lead to selection bias which could affect the results of the study. Furthermore, the limited number of graduates from rural background in this study limits the drawing of valid conclusions for this subgroup. This also makes it impossible to compare the results of this study with well-established evidence internationally that rural background is associated with willingness to practice in remote areas and choice of specialty after graduation. This is supported by findings of a considerable number of international studies [12–15]. This is also a cross-sectional study that makes it difficult to determine the direction of the association. The findings of this study should also be discussed in light of the fact that we did not collect any data on the socio-economic status of the parents/guardians of the graduates.

Further studies should thus be done with larger cohorts of graduates with rural background in Ghana. Likewise, the fact that there were no female graduates of rural background made it difficult to assess the willingness of female graduates of rural background to practice in remote areas and make a comparison with their male counterparts.

## Conclusion

The majority of graduates from the towns and cities in Ghana, with a male predominance, indicated that COBES influenced their choice of specialty and willingness to practice in the rural areas despite their town or city based upbringing. Based on these findings, it is recommended that the Ghana MoH could consider initiating pilot interventions especially educational reforms focusing on COBES in addressing the disparity of health professionals in the remote areas of the country.

## Additional file

Additional file 1: University for development studies. (DOCX 16 kb)

## Abbreviations

COBES: Community-Based Education and Service
PBL: Problem-Based Learning
UDS: University for Development Studies
SMHS: School of Medicine and Health Sciences
MDG: Millennium Development Goals
MOH: Ministry of Health
LAMICs: Low-and-Middle-Income Countries
UNDP: United Nations Development Programme
