# Supplementary material for: The effect of Community Based Education and Service (COBES) on medical graduates’ choice of specialty and willingness to work in rural communities in Ghana
Source: BMC Med Educ. 2016 Mar 1;16:79. doi: 10.1186/s12909-016-0602-8 (PMC4774102; doi:10.1186/s12909-016-0602-8)
Supplement: Additional file 1: — University for development studies. (DOCX 16 kb) [file 12909_2016_602_MOESM1_ESM.docx]

**UNIVERSITY FOR DEVELOPMENT STUDIES**

SCHOOL OF MEDICINE AND HEALTH SCIENCES

PBL OFFICE

**EXIT QUESTIONNAIRE FOR PIONERING GRADUATES OF PBL/COBES CURRICULUM**

**Introduction**

The School of Medicine and Health Sciences (SMHS) of the University for Development Studies (UDS), Ghana, successfully adopted its traditional medical training curriculum to Problem-Based Learning (PBL) and Community-Based Education and Service (COBES) methodology in 2007. COBES describes those “learning activities that use the community extensively as a learning environment, in which not only students but also teachers, members of the community, and representatives of other sectors are actively engaged throughout the educational experience” (World Health Organization, WHO, 1987). COBES component of the PBL/COBES curriculum of UDS-SMHS is the process by which teaching and learning is done in the community. As pioneer Graduates (first batch of students) of the PBL/COBES programme we would be grateful if you could spend some time to complete the questionnaire on your experiences of COBES in the communities.

**Demographic characteristics**

1. What is your age: …………………………..

1. Gender 1. Male 2. Female
2. Where were you living before entering medical school? (Tick)
3. Village
4. Town

3. City (NB. The cities in Ghana are Accra, Kumasi, Takoradi/Sekondi and Tamale)

4. Do you think COBES is useful in the study of medicine? 1. Yes 2. No

5. If yes to 4 above, what are your experiences with COBES regarding its usefulness in the study of medicine?

----------------------------------------------------------------------------------------------------------------------------------------------------------------------------------------------------------------------------------------------------------------------------------------------------------------------------------------------------------------------------------------------------------------------------

6. Do you think your experience could affect your choice of specialty in future? 1. Yes 2. No 3. Other-

7. If yes to 6 above, how will your experiences in the community through COBES affect your choice of specialty?

----------------------------------------------------------------------------------------------------------------------------------------------------------------------------------------------------------------------------------------------------------------------------------------------------------------------------------------------------------------------------------------------------------------------------

8. Do you think your experience through COBES could influence your choice of practice in a rural location? 1. Yes 2. No 3. Other

9. If yes to 8 above, how will your experience in the community through COBES affect your practice location in a rural location?

-------------------------------------------------------------------------------------------------------------------------------------------------------------------------------------------------------------------------------------------------------------------------------------------------------------------------------------------------------------------------------------------------------------------------

10. Do you think the communities in which you undertake COBES benefit in any way from the programme? 1. Yes 2. No

11. If yes to 10 above, in what ways is COBES beneficial to the community?

----------------------------------------------------------------------------------------------------------------------------------------------------------------------------------------------------------------------------------------------------------------------------------------------------------------------------------------------------------------------------------------------------------------------------

12. What are impressions regarding the organization of COBES by the medical school?--------------------------------------------------------------------------------------------------------------------------------------------------------------------------------------------------------------------------------------------------------------------------------------------------------------------------------------------

13. What could be done to improve students’ experience of COBES?----------------------------------------------------------------------------------------------------------------------------------------------------------------------------------------------------------------------------------------------------------------------------------------------------------------------------------------------------------------------------

14. What would in your opinion encourage students to want to choose a rural placement?

----------------------------------------------------------------------------------------------------------------------------------------------------------------------------------------------------------------------------------------------------------------------------------------------------------------------------------------------------------------------------------------------------------------------------

I APPRECIATE YOUR TIME SPENT TO COMPLETE THIS QUESTIONNAIRE. THANKS
